# Supplementary material for: Estimating enteric fever seroincidence in rural western Cambodia: Findings from a population-based cross-sectional serosurvey
Source: PLoS Negl Trop Dis. 2026 Sep 2;20(9):e0014691. doi: 10.1371/journal.pntd.0014691 (PMC13561420; doi:10.1371/journal.pntd.0014691)
Supplement: Supplementary file 1 [file pntd.0014691.s001.docx]

**S1 Table. Seroincidence estimated by IgA or IgG alone, overall, by age group and district.**

| **Characteristics** | **Overall*** | **Koas Krala*** | **Pailin*** | **Rukhakiri*** | **Samlout*** |
| --- | --- | --- | --- | --- | --- |
| **IgA** | | | | | |
| Overall | 156.5 (136.8, 179) | 154.4 (116.5, 204.7) | 174.5 (136.3, 223.5) | 134.8 (88.5, 205.2) | 152.3 (122.6, 189.2) |
| 5-15 years | 127.7 (100.4, 162.4) | 99.8 (63.8, 156.3) | 275.9 (144.1, 528.5) | 144.6 (71.5, 292.5) | 97.4 (70.9, 133.9) |
| 16-25 years | 167.6 (143.9, 195.2) | 178 (130, 243.8) | 153.9 (116.1, 203.9) | 132.5 (81.5, 215.5) | 182.7 (141.6, 235.7) |
| **IgG** | | | | | |
| Overall | 165.2 (149.2, 182.9) | 257 (213.3, 309.7) | 149.8 (125.1, 179.4) | 180.4 (144.5, 225.1) | 132.5 (110.9, 158.4) |
| 5-15 years | 157.7 (139.4, 178.4) | 258.7 (206.1, 324.7) | 134.2 (106.2, 169.6) | 194.7 (152.6, 248.6) | 120.6 (96.6, 150.4) |
| 16-25 years | 189.8 (164.4, 219.3) | 251 (188.7, 334) | 201.7 (152.6, 266.7) | 135.3 (97.3, 188) | 168.1 (134.6, 210) |
| *Seroincidence (95%CI), all reported per 1,000 person-years | | | | | |
