# Supplementary material for: Estimating enteric fever seroincidence in rural western Cambodia: Findings from a population-based cross-sectional serosurvey
Source: PLoS Negl Trop Dis. 2026 Sep 2;20(9):e0014691. doi: 10.1371/journal.pntd.0014691 (PMC13561420; doi:10.1371/journal.pntd.0014691)
Supplement: Supplementary file 2 [file pntd.0014691.s002.docx]

**S2 Table. Comparisons of enteric fever seroincidence using two-sample z-test, by district, age group, WASH access, household wealth status, and month of data collection.**

| **Variable** | **Comparison group** | **Seroincidence differences (95%CI)*** | **z statistic** | **p-value** |
| --- | --- | --- | --- | --- |
| **District** |  |  |  |  |
|  | Koas Krala vs.Samlout | 69.6 (23.9, 115.3) | 2.983993 | 0.002845 |
|  | Koas Krala vs.Pailin | 50.9 (2.2, 99.6) | 2.048478 | 0.040513 |
|  | Koas Krala vs.Rukhakiri | 47.9 (-7.1, 102.9) | 1.705782 | 0.088049 |
|  | Pailin vs.Rukhakiri | -3 (-49.2, 43.2) | -0.12662 | 0.899238 |
|  | Pailin vs.Samlout | 18.7 (-15.9, 53.3) | 1.061547 | 0.288441 |
|  | Rukhakiri vs.Samlout | 69.6 (23.9, 115.3) | 0.988237 | 0.323037 |
| **Age group** |  |  |  |  |
|  | 16-25 years vs. 5-15 years | -18.5 (-48, 10.9) | -1.23338 | 0.217434 |
| Age group by district |  |  |  |  |
| Koas Krala | 16-25 years vs. 5-15 years | -80.9 (-154.3, -7.5) | -2.16105 | 0.030691 |
| Rukhakiri | 16-25 years vs. 5-15 years | -45.5 (-119.1, 28.2) | -1.21073 | 0.225999 |
| Pailin | 16-25 years vs. 5-15 years | 55.6 (-17.1, 128.4) | 1.499515 | 0.13374 |
| Samlout | 16-25 years vs. 5-15 years | -22.9 (-61.3, 15.6) | -1.16605 | 0.243593 |
| **WASH access** |  |  |  |  |
|  | ≥1 unimproved vs. Both improved | 1.8 (-29.1, 32.7) | 0.114096 | 0.909162 |
| WASH access by district |  |  |  |  |
| Koas Krala | ≥1 unimproved vs. Both improved | 19.6 (-77.6, 116.8) | 0.395702 | 0.692325 |
| Rukhakiri | ≥1 unimproved vs. Both improved | 3.8 (-80.8, 88.4) | 0.088783 | 0.929254 |
| Pailin | ≥1 unimproved vs. Both improved | -1.3 (-56, 53.3) | -0.04781 | 0.961864 |
| Samlout | ≥1 unimproved vs. Both improved | -25.3 (-67.2, 16.5) | -1.18529 | 0.235902 |
| WASH access by age group |  |  |  |  |
| 16-25 years | ≥1 unimproved vs. Both improved | -39.5 (-92.2, 13.3) | -1.46658 | 0.14249 |
| 5-15 years | ≥1 unimproved vs. Both improved | 15.9 (-20.9, 52.7) | 0.848569 | 0.396121 |
| **Household wealth status** |  |  |  |  |
|  | Poor vs. Middle | 19.9 (-24.1, 63.9) | 0.88656 | 0.375316 |
|  | Poor vs. Rich | 16.7 (-22, 55.3) | 0.845943 | 0.397585 |
|  | Middle vs. Rich | -3.2 (-38.3, 31.9) | -0.18056 | 0.856716 |
| Household wealth status by district |  |  |  |  |
| Koas Krala | Poor vs. Middle | -0.3 (-117.5, 116.8) | -0.00528 | 0.995787 |
|  | Poor vs. Rich | -3.9 (-86.3, 78.5) | -0.09293 | 0.925958 |
|  | Middle vs. Rich | -3.6 (-118.1, 110.9) | -0.06148 | 0.950974 |
| Rukhakiri | Poor vs. Middle | -5.5 (-101.6, 90.7) | -0.11136 | 0.911327 |
|  | Poor vs. Rich | 48.4 (-51.8, 148.6) | 0.946939 | 0.34367 |
|  | Middle vs. Rich | 53.9 (-17.4, 125.2) | 1.481738 | 0.13841 |
| Pailin | Poor vs. Middle | 69.4 (-17.5, 156.3) | 1.566204 | 0.117301 |
|  | Poor vs. Rich | 59.4 (-16.5, 135.4) | 1.533133 | 0.125243 |
|  | Middle vs. Rich | -10 (-72.6, 52.6) | -0.31315 | 0.754166 |
| Samlout | Poor vs. Middle | -5.5 (-101.6, 90.7) | -0.11136 | 0.911327 |
|  | Poor vs. Rich | 48.4 (-51.8, 148.6) | 0.946939 | 0.34367 |
|  | Middle vs. Rich | 53.9 (-17.4, 125.2) | 1.481738 | 0.13841 |
| Household wealth status by age group |  |  |  |  |
| 16-25 years | Poor vs. Middle | -10.7 (-64.7, 43.3) | -0.38823 | 0.697849 |
|  | Poor vs. Rich | -32.9 (-85.3, 19.5) | -1.22947 | 0.218896 |
|  | Middle vs. Rich | -22.2 (-75.6, 31.2) | -0.81405 | 0.415617 |
| 5-15 years | Poor vs. Middle | 27.7 (-24.3, 79.7) | 1.045087 | 0.295983 |
|  | Poor vs. Rich | 31.1 (-14.1, 76.2) | 1.348865 | 0.17738 |
|  | Middle vs. Rich | 3.3 (-38.5, 45.1) | 0.155864 | 0.87614 |
| **Month of data collection** |  |  |  |  |
|  | Apr, May vs. Aug | -180.5 (-260.7, -100.3) | -4.40917 | 1.04E-05 |
|  | Apr, May vs. Jun | 39.4 (3.4, 75.5) | 2.142398 | 0.032161 |
|  | Apr, May vs. Oct, Nov | 0.6 (-38.6, 39.9) | 0.030985 | 0.975282 |
|  | Apr, May vs. Sep | 2.1 (-36.3, 40.5) | 0.106626 | 0.915085 |
|  | Aug vs. Jun | 219.9 (140.4, 299.4) | 5.422816 | 5.87E-08 |
|  | Aug vs. Oct, Nov | 181.1 (100.1, 262.1) | 4.383039 | 1.17E-05 |
|  | Aug vs. Sep | 182.6 (102, 263.1) | 4.442324 | 8.9E-06 |
|  | Jun vs. Oct, Nov | -38.8 (-76.5, -1.1) | -2.01628 | 0.043771 |
|  | Jun vs. Sep | -37.3 (-74.1, -0.6) | -1.98971 | 0.046623 |
|  | Oct, Nov vs. Sep | 1.5 (-38.5, 41.4) | 0.071992 | 0.942609 |
| *Seroincidence differences (95%CI) reported per 1,000 person-years | | | | |
