# Supplementary material for: Estimating enteric fever seroincidence in rural western Cambodia: Findings from a population-based cross-sectional serosurvey
Source: PLoS Negl Trop Dis. 2026 Sep 2;20(9):e0014691. doi: 10.1371/journal.pntd.0014691 (PMC13561420; doi:10.1371/journal.pntd.0014691)
Supplement: Supplementary file 3 [file pntd.0014691.s003.docx]

**S3 Table. HLyE IgA and IgG seroincidence by month of data collection**

| **Data collection period *** | **Number of participants** | **Seroincidence (95%CI) / 1,000 person-years^** |
| --- | --- | --- |
| Apr, May† | 137 | 150.7 (126.2, 179.8) |
| Jun | 70 | 111.2 (89.4, 138.4) |
| Aug | 96 | 331.1 (263.5, 416.2) |
| Sep | 118 | 148.6 (123.4, 178.9) |
| Oct, Nov† | 108 | 150 (123.8, 181.8) |

* Sample collection occurred during 8 months of the recruitment period (all activities paused in July due to local political activities, and no samples were collected in December). The data collection months of each district are: Rukhakiri- April (n=46), May (n=8); Koas Krala- August (n=67), September (n=69); Samlout- May (n=83), June (n=70), August (n=29), November (n=16); Pailin- September (n=49), October (n=92).

† Data from April and May were combined, as were the months October and November, due to the small number of participants recruited in April and November.

^Seroincidence differed significantly by month of data collection, with August showing higher estimates than all other months (p<0·001), and June differing significantly from April, May, September, and October, November (p<0·05).
